# Current Concepts on Antiplatelet Therapy: Focus on the Novel Thienopyridine and Non-Thienopyridine Agents

**DOI:** 10.1155/2010/595934

**Published:** 2010-12-05

**Authors:** L. Testa, G. G. L. Biondi Zoccai, M. Valgimigli, R. A. Latini, S. Pizzocri, S. Lanotte, M. L. Laudisa, N. Brambilla, M. R. Ward, G. A. Figtree, F. Bedogni, R. Bhindi

**Affiliations:** ^1^Interventional Cardiology Department, St. Ambrogio Clinical Institute, 20149, Milan, Italy; ^2^Institute of Cardiology, Ospedale “Le Molinette”, University of Turin, 10124, Turin, Italy; ^3^Department of Cardiology, Arcispedale S. Anna, University of Ferrara, 44100, Ferrara, Italy; ^4^Department of Cardiology, Royal North Shore Hospital, North Shore Heart Research Group, Kolling Institute, University of Sydney, Sydney NSW 2065, Australia

## Abstract

Thienopyridines are a class of drug targeting the platelet adenosine diphosphate (ADP) 2 receptor. They significantly reduce platelet activity and are therefore clinically beneficial in settings where platelet activation is a key pathophysiological feature, particularly myocardial infarction. Ticlopidine, the first of the class introduced to clinical practice, was soon challenged and almost completely replaced by clopidogrel for its better tolerability. More recently, prasugrel and ticagrelor have been shown to provide a more powerful antiplatelet action compared to clopidogrel but at a cost of higher risk of bleeding complications. Cangrelor, a molecule very similar to ticagrelor, is currently being evaluated against clopidogrel. Considering the key balance of ischemic protection and bleeding risk, this paper discusses the background to the development of prasugrel, ticagrelor, and cangrelor and aims to characterise their risk-benefit profile and possible implementation in daily practice.

## 1. Introduction

Ticlopidine, clopidogrel, and more recently prasugrel, constitute the class of drugs called thienopyridines which is characterized by the selective targeting of the adenosine diphosphate (ADP) 2 receptor on the surface of platelets. Blockage of this receptor results in reduced platelet activation which may have critical clinical benefits in settings where platelet activation is a key pathophysiological feature such as in myocardial infarction. Historically, ticlopidine was the first member of this class. However, its not insignificant, risk of neutropenia and thrombotic thrombocytopenic purpura led to its predominant replacement by clopidogrel in routine clinical practice [1]. 

Thienopyridines play a key role in the cardiovascular arena. Their application covers the wide spectrum of patients with stable angina (SA), acute coronary syndromes (ACSs), and/or those undergoing percutaneous coronary intervention (PCI). In these settings they reduce the restenosis rate, the risk of thrombosis and major adverse cardiac events [2, 3]. Perhaps clopidogrel administration alone is advisable when there is contraindication to or aspirin intolerance [4]. In acute myocardial infarction, for patients who have undergone diagnostic cardiac catheterization and for whom PCI is planned, clopidogrel should be started and continued for at least 1 month after bare metal stent implantation and for several months after drug-eluting stent implantation up to 12 months in patients who are not at high risk for bleeding [5]. In patients with unstable angina, clopidogrel should be started on admission and administered for at least 1 month, and, according to many experts, up to 9 months [6]. Overall, for those patient treated with PCI the coadministration of aspirin (permanently) and clopidogrel is mandatory. Following a bare metal or a drug eluting coronary stent implantation, clopidogrel is administered for at least 1 month and 1 year, respectively [7].

On the basis of *ex vivo *platelet function tests [8–12], it has been recently reported that up to 34% of patients show a suboptimal antiplatelet response to clopidogrel (“clopidogrel nonresponders”) and, according to some pivotal studies [13–15], these individuals should be considered as having a higher risk of adverse clinical events in acute as well as chronic coronary artery disease. This evidence drove the search for more efficacious and reliable antiplatelet therapies. 

A new thienopyridine agent Prasugrel (Effient, Eli Lilly, Indianapolis, IN) [16], and two cyclopentyl triazolo pyrimidines agents, ticagrelor (Brilinta, AstraZeneca, Wilmington, DL) [17], and cangrelor (Cangrelor, The Medicines Company, Parsippany, NJ) [18, 19] have been introduced, aimed at overcoming the drawbacks of their predecessors such as variable antiplatelet efficacy and risk of side effects. Only the first has received approval from the FDA and the EMEA [20].

This paper will elucidate the evolution and possible clinical application of these novel antiplatelet agents.

## 2. Mechanism of Action of Prasugrel, Ticagrelor, and Cangrelor

**Figure 1:**
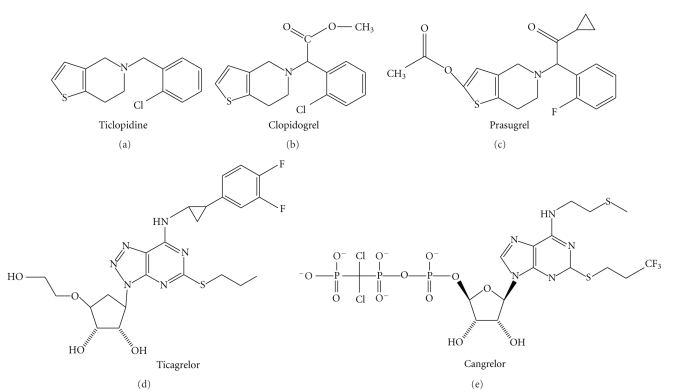
Biochemical structures of thienopyridine and nonthienopirydine antiplatelet agents.

Like other members of the thienopyridine class (Figure 1), Prasugrel is a prodrug absorbed via the gut and is hydrolysed into a thiolactibe (R-95913) via esterases in the gut wall, liver, and plasma. This is then oxidised into the active thiol metabolite R-138727 by the cytochrome p450 system, mainly through the CYP3A and CYP2B6 isoenzymes. The latter irreversibly binds the G-protein linked P2Y_12_ ADP receptors on the platelet surface. An important difference between the metabolism of prasugrel and clopidogrel is that a significant portion of the administered dose of clopidogrel is deactivated in the early stages of its metabolism, resulting in less availability of the active metabolite.

Ticagrelor (AZD6140) is the first member of a new class of orally active antiplatelet agents not requiring metabolic activation. Ticagrelor is essentially an adenosine-triphosphate (ATP) derivate (see Figure 1). Like the thienopyridine class of drugs, it inhibits the P2Y_12 _ADP-receptor, however, this inhibitory linkage is completely reversible. 

Like ticagrelor, cangrelor has been derived from ATP (see Figure 1) and, at present, it can be administered only intravenously. After infusion, it reversibly binds the P2Y_12_ receptor with high affinity without requiring a metabolic activation.

## 3. Preclinical Studies

The key pharmacodynamic and pharmacokinetic features of the new antiplatelets agents are summarised in Table 1. Studies in several animal models show that orally administered prasugrel is completely absorbed from the gut and then rapidly metabolised in the liver to the active thiol metabolite with no selective tissue uptake. This active hepatic metabolite has a stereoselective effect and exerts a more potent and dose-dependent inhibition of platelet activity compared to ticlopidine and clopidogrel. Time from ingestion to the maximal concentration of the active metabolite is about 30 minutes. Mean elimination half-life of the active metabolite is 3.7 hours. Like other thienopyridines, the metabolites of prasugrel are excreted via the kidney [21–28].

Ticagrelor, the first studied member of the class of cyclopentyl triazolo pyrimidines, has been obtained by the beta-gamma-methylene substitution of the ester linkage in the triphosphate group of ATP. In contrast to natural ATP which is rapidly inactivated by soluble nucleotidases, it is relatively resistant to enzymatic degradation. Moreover, it does not require any metabolic activation although an active metabolite has been identified with quite similar features and antiplatelet capacity thus participating in the overall antiplatelet effect. Peak plasma level of ticagrelor is reached after 1.5–3 hours and the half-life is 6–12 hours, depending on the dose [29, 30] and more than 70% is excreted via the kidney. 

In animal models, cangrelor inhibits *ex vivo* ADP-induced platelet aggregation without prolonging the bleeding times perhaps showing a fast restoration of platelet reactivity at the end of the infusion. It rapidly reaches the steady state concentration with a half-life of 2.6–3.3 minutes [31, 32] and it is excreted via the kidney.

## 4. Clinical Studies

### 4.1. Prasugrel

Prasugrel has been extensively investigated in several phase II studies consistently showing that concentration of the active prasugrel metabolite are higher, both acutely and after 2 weeks of treatment, than the active clopidogrel metabolite thus supporting the hypothesis that the observed differences in pharmacodynamic effects could be due to differences in metabolic efficiency in generating the respective metabolites [33, 34]. 

The Joint Utilisation of Medications to Block Platelets Optimally (JUMBO-TIMI 26) study reported an equivalent risk of major bleeding events compared to clopidogrel thus providing important safety data which facilitated further evaluation of efficacy of prasugrel [35, 36]. 

The large phase 3 Trial to Assess Improvement in Therapeutic Outcomes by Optimizing Platelet Inhibition with Prasugrel-Thrombolysis in Myocardial Infarction (TRITON-TIMI) 38 trial [37] was a double-blinded study that randomised 13608 patients with acute coronary syndromes to aspirin and prasugrel (60 mg loading dose followed by 10 mg daily) or aspirin and clopidogrel (300 mg loading dose followed by 75 mg daily). The composite endpoint of cardiovascular death/myocardial infarction/stroke was significantly reduced in the prasugrel group, largely driven by a lower rate of nonfatal myocardial infarction. No benefit in terms of mortality was observed while a significant increase in bleeding complications in the prasugrel group was reported. According to subgroup analyses, patients with diabetes mellitus, myocardial infarction, or complex coronary lesions benefited most from prasugrel treatment. On the other hand, elderly patients, patients with a low body weight (<60 kg) or a previous history of stroke or transient ischemic attacks did not benefit [37]. 

In a recently published substudy (TRITON TIMI 38 substudy) which included 12844 patients undergoing coronary stent implantation (bare metal or drug eluting stent), prasugrel was associated with a significantly reduced risk of ischemic events and stent thrombosis, compared to clopidogrel [38]. 

In a TRITON-TIMI 38 substudy, clinical endpoints in the acute phase (within 3 days) and from the third day till the end of followup have been compared. Of note, the loading as well as the maintenance dose of prasugrel were superior to clopidogrel for the reduction of ischemic events [39]. Moreover, the excess of bleeding in the former group was related to the maintenance dose only. Recently, it has been shown that Prasugrel is able to provide a net benefit over clopidogrel particularly in patients with diabetes mellitus, that is, a reduction of ischemic events not counterbalanced by an increased risk of major bleedings [40]. 

Prasugrel has also been shown to be superior to clopidogrel in the setting of ST elevation myocardial infarction with a durable benefit up to 15 months [41]. 

On a pharmacodynamic/pharmacologic basis such a superiority might be explained by the absence of any interaction of the common functional cytochrome P450 genetic variants with drug metabolite levels and inhibition of platelet aggregation [42]. 

We have recently showed, by means of an explorative meta-analysis, that prasugrel compared to clopidogrel is associated with a one-third higher risk and a two-fold higher risk of major and minor bleedings, respectively [20].

### 4.2. Ticagrelor

The Dose Confirmation Study assessing antiplatelet Effects of AZD6140 versus clopidogRel in non-ST segment Elevation myocardial infarction (DISPERSE-2) study [43] enrolled 990 patients admitted with non-ST elevation acute coronary syndromes. Patients were allocated, in a randomised fashion, to 300 mg loading dose of clopidogrel followed by 75 mg daily for 3 months versus ticagrelor 90 mg twice daily for 3 months or 180 mg twice daily for 3 months. Twenty-five percent of the patients were diabetic while a diagnosis of previous myocardial infarction was present in about 24% of the cases. With respect to the primary endpoint, the cumulative rate of major and minor bleedings, no significant differences were found between the two doses of ticagrelor versus clopidogrel while a trend in favour of ticagrelor was seen according to the risk of myocardial infarction. 

The Platelet Inhibition and Patient Outcomes (PLATO) trial [17] was a large multicenter randomised controlled trials that randomised more than 18000 patients with ST elevation as well as non-ST-elevation acute coronary syndromes. Patients were allocated to either clopidogrel 300 mg (600 mg in 19.6% of the cases) loading, then 75 mg daily for 9 months or ticagrelor 180 mg loading, then 90 mg twice daily for 9 months. Twenty-five percent of the patients were diabetic while a diagnosis of previous myocardial infarction was present in about 21% of the cases. Ticagrelor was associated with a significantly lower risk of the primary endpoint (composite of cardiovascular death, myocardial infarction, or stroke) while no differences were seen in the rates of major bleeding.

### 4.3. Cangrelor

The CHAMPION PCI study [18] was a randomized, double-blind, double-dummy, active-control trial comparing cangrelor with 600 mg of clopidogrel in patients with acute coronary syndromes undergoing PCI. Within 30 minutes of PCI, all patients received either cangrelor (in an intravenous bolus of 30 *μ*g per kilogram of body weight and an intravenous infusion of 4 *μ*g per kilogram per minute) or a placebo bolus and infusion. Patients received 600 mg of clopidogrel (in four 150-mg capsules) or placebo at the time of infusion. To allow the transition from intravenous cangrelor to oral clopidogrel, patients received another four capsules (either clopidogrel in patients receiving cangrelor or placebo in patients receiving clopidogrel) at the discontinuation of the study drug infusion. 

At 48 hours as well as at 30 days, cangrelor was not superior to clopidogrel with respect to the primary composite endpoint (death from any cause, myocardial infarction, or ischemia-driven revascularization) while, with respect to the risk of major haemorrhage, cangrelor was associated with a non statistically significantly higher risk.

In the CHAMPION PLATFORM [19], patients underwent randomization according to a double-blind, placebo-controlled, double-dummy design to receive either cangrelor (bolus of 30 *μ*g per kilogram of body weight followed by infusion of 4 *μ*g per kilogram per minute) or a placebo bolus and infusion “for the duration of the PCI procedure”, with a minimum infusion duration of 2 hours and a maximum of 4 hours. Patients in the cangrelor group received 600 mg of clopidogrel after the end of the cangrelor infusion, and those in the placebo group received 600 mg of clopidogrel at the end of the procedure. Cangrelor was not superior to placebo in reducing the primary endpoint (death, myocardial infarction, or ischemia-driven revascularization at 48 hours). The prespecified secondary endpoints of stent thrombosis and death were lower in the cangrelor group, with no significant increase in the rate of transfusion. 

These two trials have been conducted and published in parallel. Of note, at the 70% interim analysis, the interim-analysis review committee reported that the estimated conditional power of CHAMPION PCI to demonstrate superiority was low. However, in the absence of safety concerns, the executive committee and sponsor elected to continue the CHAMPION PCI trial until the CHAMPION PLATFORM underwent its 70% interim analysis. At that time, the interim-analysis review committee and the data and safety monitoring board reported that the estimated conditional power in CHAMPION PLATFORM was also low and recommended discontinuation of enrolment into both trials. 

Overall, these two trials do not support the implementation of cangrelor in routine practice, however, a third trial (BRIDGE: Maintenance of Platelet inihiBition With cangRelor After dIscontinuation of ThienopyriDines in Patients Undergoing surgery, http://clinicaltrials.gov/ct2/show/NCT00767507) is still ongoing aiming at investigating the possible protective role of cangrelor in patients who discontinue antiplatelet agents waiting for heart surgery.

### 4.4. Are Prasugrel and Ticagrelor Ready for Prime Time?

Being more potent and reliable in its antiplatelet effect, when administered in combination with aspirin to patients with ACS undergoing PCI, prasugrel reduced both ischemic coronary events and the incidence of coronary stent thrombosis compared with the combination of aspirin and clopidogrel. 

More powerful antiplatelet activity comes at a cost of higher risk of major bleeding, thus an accurate selection of those patients with a favourable risk/benefit profile is mandatory. This selection is even more important when considering that it has been compared against clopidogrel in an unselected population with respect to clopidogrel responsiveness. The assessment of the latter would imply a consensus over the right tool to measure the residual platelet activity and the exact timing. Moreover, in order to standardise the bleeding risk it should be consensually adopted one of the several bleeding risk score currently available [44–47]. 

Prasugrel has already received approval from EMEA and FDA [20] and it is clearly a very promising drug but, consistent with any other new tool, a reasonable enthusiasm should always be balanced with objective analyses.

Ticagrelor was associated with a much higher incidence of dyspnea as compared with clopidogrel in PLATO (14.2% of patients versus 9.2%; *P* < 0.001), something that had been observed in earlier phase 2 trials with the investigational agent. 

However, most episodes lasted less than a week and discontinuation of the study drug because of dyspnea occurred in 0.9% of patients in the ticagrelor group. In terms of other side effects, Holter monitoring did detect more frequent ventricular pauses during the first week in the ticagrelor group than in the clopidogrel group, but such episodes were infrequent at 30 days and were rarely associated with symptoms. There were no significant differences in the rates of clinical manifestations of bradyarrhythmia between the two treatment groups.

Ticagrelor has been defined as a prospective “blockbuster drug”: in 2009, clopidogrel was the second-biggest-selling drug in the world, with global revenues of more than $8 billion although generic versions of clopidogrel are already available in Europe and are expected to appear in the US within a few years. Considering the high number of patients “clopidogrel nonresponders”, these people likely represent the initial target for newer antiplatelet drugs. 

The potential availability of three agents, clopidogrel, prasugrel, and ticagrelor, for antagonizing platelet ADP receptors would probably give a real chance to tailor antiplatelet therapy.

A specific setting in which ticagrelor may be preferred could be in patients whose coronary anatomy is unknown and for whom a CABG procedure is deemed probable. And if patients who are receiving clopidogrel or prasugrel need elective surgery, it would seem reasonable to switch them to ticagrelor five to seven days before surgery because of its reversible platelet inhibition. 

However, like prasugrel, the use of ticagrelor should probably be avoided in those with a history of stroke or transient ischemic attack and in those with a high risk of bleeding. Given concerns over dyspnea, ticagrelor should also be avoided in those with chronic obstructive pulmonary disease, bradyarrhythmias unprotected by pacemakers or a history of syncope. 

Lastly, for all remaining patients with ACS, either ticagrelor or prasugrel might be the right option.

While waiting for specifically designed trials, data from indirect comparison of prasugrel and ticagrelor showed that similar efficacy and safety have to be acknowledged, but prasugrel appeared more protective from coronary stent thrombosis, while causing more bleedings [48].

## 5. Conclusion

The introduction of generic clopidogrel will probably make clinical decision making even more complicated. In the world of multiple new antiplatelet agents, considering both the need for ischemic protection and the hazard of bleeding will be crucial.

## Figures and Tables

**Table 1:** Main pharmacodynamic/pharmacokinetic features of novel antiplatelet agents.

	Family	Admin. route	Need for metabolic activation	Active metabolite	Linkage reversibility	Half life (h)	Excretion
Prasugrel	thienopyridines	Oral	Yes	Yes	No	3.7	Kidney
Ticagrelor	Cyclopentyltria olo-pyrimidines	Oral	No	Yes	Yes	6–12	Kidney
Cangrelor	Cyclopentyltria olo-pyrimidines	Intravenous	No	No	Yes	2.6–3.3	Kidney
